# Clinical validation of the “Straight-Leg-Evaluation-Trauma-Test” (SILENT) as a rapid assessment tool for injuries of the lower extremity in trauma bay patients

**DOI:** 10.1007/s00068-023-02437-z

**Published:** 2024-01-23

**Authors:** Till Berk, Valentin Neuhaus, Catalina Nierlich, Zsolt J. Balogh, Felix Karl-Ludwig Klingebiel, Yannik Kalbas, Hans-Christoph Pape, Sascha Halvachizadeh

**Affiliations:** 1 Department of Trauma, University Hospital Zurich, Raemistrasse 100, 8091 Zurich, Switzerland; 2 Faculty of Medicine, University of Zurich, Raemistrasse 71, 8006 Zurich, Switzerland; 3 Harald-Tscherne Laboratory for Orthopedic and Trauma Research, University of Zurich, Sternwartstrasse 14, 8091 Zurich, Switzerland; 4 Department of Traumatology, John Hunter Hospital and University of Newcastle, Newcastle, NSW Australia

**Keywords:** SILENT test, Trauma bay, ATLS, Physical examination, Primary survey

## Abstract

**Purpose:**

Clinical assessment of the major trauma patient follows international validated guidelines without standardized trauma-specific assessment of the lower extremities for injuries. This study aimed to validate a novel clinical test for lower extremity evaluation during trauma resuscitation phase.

**Methods:**

This diagnostic, prognostic observational cohort study was performed on trauma patient treated at one level I trauma center between Mar 2022 and Mar 2023. The Straight-Leg-Evaluation-Trauma (SILENT) test follows three steps during the primary survey: inspection for obvious fractures (e.g., open fracture), active elevation of the leg, and cautious elevation of the lower extremity from the heel. SILENT was considered positive when obvious fracture was present and painful or pathological mobility was observed. The SILENT test was compared with standardized radiographs (CT scan or X-ray) as the reference test for fractures. Statistical analysis included sensitivity, specificity, and receiver operating characteristic testing.

**Results:**

403 trauma bay patients were included, mean age 51.6 (SD 21.2) years with 83 fractures of the lower extremity and 27 pelvic/acetabular fractures. Overall sensitivity was 75% (95%CI 64 to 84%), and overall specificity was 99% (95%CI 97 to 100%). Highest sensitivity was for detection of tibia fractures (93%, 95%CI 77 to 99%). Sensitivity of SILENT was higher in the unconscious patient (96%, 95%CI 78 to 100%) with a near 100% specificity. AUC was highest for tibia fractures (0.96, 95%CI 0.92 to 1.0) followed by femur fractures (0.92, 95%CI 0.84 to 0.99).

**Conclusion:**

The SILENT test is a clinical applicable and feasible rule-out test for relevant injuries of the lower extremity. A negative SILENT test of the femur or the tibia might reduce the requirement of additional radiological imaging. Further large-scale prospective studies might be required to corroborate the beneficial effects of the SILENT test.

**Supplementary Information:**

The online version contains supplementary material available at 10.1007/s00068-023-02437-z.

## Introduction

The initial assessment of major trauma patients follows international standardized and validated protocols [1, 2]. According to the ATLS® guidelines, the initial evaluation of the major trauma patients (primary survey) includes the detection of life-threatening injuries [2]. The treatment of major trauma patients is time sensitive and the trauma team is required to rule out life-threatening injuries as quickly and as precisely as possible. The trauma team examines the chest, the abdomen, the pelvis, and the femur for life-threatening bleeding sources. Following the initial clinical assessment, the trauma team decides whether the patient qualifies for a trauma computerized tomography (CT) scan [3]. The routine trauma CT scan in major trauma patients includes the CT scan of the head, the entire spinal axis, the chest, the abdomen, the pelvis, and the hip joint and does not scan further than the lesser trochanter, unless the treating physician requires additional examinations [4, 5]. The number of missed injuries in major trauma patients ranges from 1.3 to 39%; out of these, 15 to 22.3% of patients had clinically significant missed injuries [6]. Missed injuries of the lower extremity have been reported to be up to 30.3% [7]. One potential reason might be the lack of standardized clinical assessments of the limbs in a trauma bay setting coupled by the fact that the extremities are not routinely included in the standard trauma CT scan [8]. The examination of the lower extremity still does not follow standardized guidelines. There is a need for a clinical feasible and quick assessment method to rule out major fractures of the lower extremity that are not included in the routine CT scan. We therefore developed a novel clinical examination that is quick and safe, and is less examiner dependent compared with routine clinical examination [9]. The StraIght-Leg-EvaluatioN-Trauma (SILENT test, also known as the Neuhaus-Pape-Berk test) is of higher certainty and higher predictive value for fractures than routine clinical testing of the lower extremities in an experimental study [9]. The aim of this study was the evaluation of clinical feasibility of the SILENT test during the primary survey of trauma patients. Our hypothesis was that the SILENT test is comparable to the clinical standard tests in detecting fractures of the lower extremity.

## Methods

The institutional ethics committee (#2022–00675) approved this diagnostic, prognostic cohort study. The study was conducted in accordance with the Declaration of Helsinki. Reporting of this study follows the STARD and TRIPOD guidelines [10].

### Participants

The SILENT test was implemented in 2022 in our clinic as a standard assessment during the initial assessment of trauma patients who were admitted to our trauma bay [3, 9]. The SILENT test was performed by trauma residents on all patients who were admitted via our trauma bay. The performing physician documented the results of the SILENT test in the admission paper. The authors developed a template that is included in the standardized documentation of the primary survey (Supplement). All data were collected prospectively. The admission via the trauma bay is based on the discretion of the prehospital medical service and follows international guidelines [1, 11]. After an implementation time of the test, all patients admitted to our trauma bay between March 1, 2022, and March 31, 2023, were consecutively included in this study. The trauma residents performed and documented the SILENT test as part of the primary survey. If the test was positive, an appropriate radiograph was performed. If SILENT was negative, no additional imaging, other than the routine trauma CT scan, was performed.

### Index test: SILENT test

The SILENT test has been published previously [9]. The clinical adoption of the SILENT test follows three steps: First, in the undressed patient, the examiner inspects the lower extremities and documents obvious fractures (e.g., open fractures, clinical visible dislocations). Second, the examiner asks the awake patient to lift one leg at a time (Halva modification). If the patient is able to actively lift the leg painfree and without any clinical signs for injuries, the SILENT test is considered negative. If the patient is not able to lift the leg, the examiner lifts the lower extremity until a maximum of 45°. The test was considered negative if no signs of potential injuries are observed.

The SILENT test is considered positive if one of the following results are observed:

- Clinical deformity or open fracture
- Pain
- Unphysiological mobility of the lower extremity

**Fig. 1:**
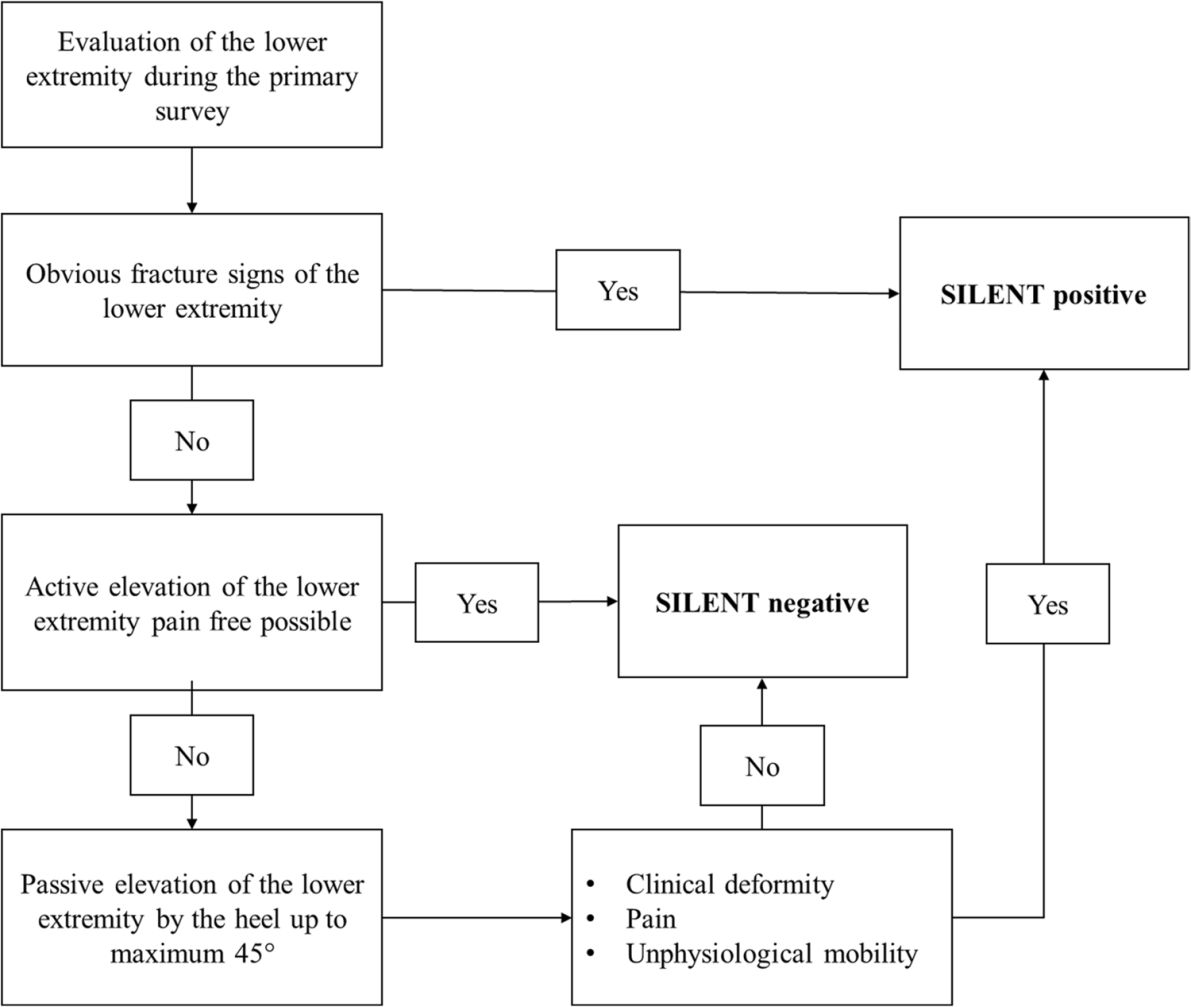
Algorithm and decision-making of the SILENT test

If the SILENT test was positive at any step, the test was not continued further (Fig. 1). The SILENT test was performed to test for pelvic and acetabular fractures, femur fractures, knee injuries, and tibia or ankle fractures.

### Reference test: radiological evaluation

A radiological imaging of the injured leg is added to the routine trauma CT scan according to the in-hospital guideline if a fracture is suspected (SILENT positive). This includes either the extension of the trauma CT scan or the performance of plain radiographs according to clinical standards. CT scan is extended at least to the adjacent joints. Additional imaging (either further extension of the CT scan or plain radiographs) depended on the clinical findings, trauma mechanism, or patient history. The radiographic image was analyzed and reported by a radiologist in real time. If the initial assessment of the trauma patients (primary, secondary survey) did not reveal a positive SILENT test, a standard clinical evaluation was additionally performed. A final clinical control was performed during the tertiary survey and a potential diagnosis of a fracture was crosschecked with the final discharge summary. If the tertiary survey was negative and the discharge papers did not document any additional fractures, the SILENT test was considered true negative. A true positive SILENT test was documented if the clinical suspicion of a fracture was confirmed radiologically. Fractures were classified according to the Arbeitsgemeinschaft für Osteosynthesefracgen/Orthopaedic Trauma Association AO/OTA classification [12].

### Analysis

Data were collected prospectively. The clinical assessment of the SILENT was documented either “positive” or “negative” for each injured area: pelvic and acetabular fractures, femur, knee, and tibia fractures. Epidemiological testing of the SILENT test includes sensitivity and specificity analyses. Demographic data are presented with mean and standard deviation (SD) for continuous variables and numbers and percentage for categorical variables. The prediction of radiologically evident fractures is presented with a receiver operating characteristic (ROC) and quantified with the area under the receiver operating curve (AUC). Patients with missing data were excluded from the analysis. A formal sample size calculation was not feasible, since there is no study evaluating clinical examination of lower extremity fractures to estimate an effect size. All analyses were performed using R version 4.2.0 (2022–04-22 ucrt) (R Core Team (2022). R: A language and environment for statistical computing. R Foundation for Statistical, Computing, Vienna, Austria. URL https://www.R-project.org/).

## Results

**Fig. 2:**
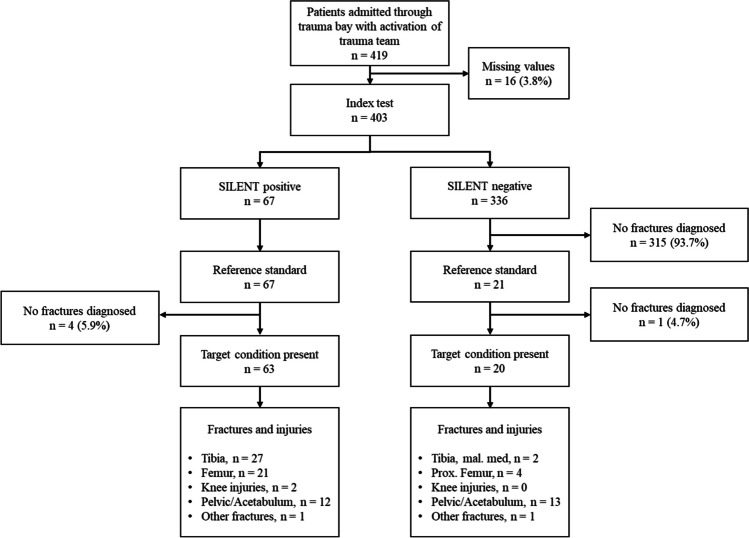
Flow chart according to TRIPOD guideline. Reference standard, radiological evaluation with either CT scan or plan x-ray according to clinical standard

This study included 403 patients with 83 fractures of the lower extremity. The index test was performed on all patients (Fig. 2). The mean age of patients was 51.6 (SD 21.2) years; 26.3% of patients were female. The most common fractures of the lower extremity were tibia fractures (34.9%) and femur fractures (30.1%) (Table 1). Most femur fractures were proximal femur fractures (60%), and besides ankle fractures (48.3%), most tibia fractures were shaft fractures (34.5%) (Table 2).

**Table 1:** Demographics of study population

	403
Female sex, *n* (%)	106 (26.3)
Age [years], mean (SD)	51.6 (21.2)
Mechanism of injury	
Motor vehicle injury, *n* (%)	140 (34.8)
Sports injury, *n* (%)	99 (24.5)
Assault or suicide, *n* (%)	18 (4.49)
Injury during work, *n* (%)	31 (7.6)
Injury at home, *n* (%)	79 (19.5)
Unknown, *n* (%)	37 (9.1)
Fractures, *n* (%)	83 (20.6)
Tibia fracture, *n* (%)	29 (34.9)
Femur fracture, *n* (%)	25 (30.1)
Hip dislocation, *n* (%)	3 (0.7)
Open fractures	
1° open fracture, *n* (%)	6 (7.2)
2° open fracture, *n* (%)	7 (8.4)
3° open fracture, *n* (%)	5 (6.0)
Other fractures and injuries, *n* (%)	32 (7.9)
Pelvic/acetabulum fracture, *n* (%)	27 (6.7)
Knee injuries, *n* (%)	3 (0.7)
Fractures of the foot, *n* (%)	2 (0.5)
ISS [points], mean (SD)	24.06 (10.34)
Intubated patients, *n* (%)	34 (8.4)

*SD*, standard deviation; *ISS*, injury severity score

**Table 2:** Distribution of femur and tibia fractures

Fracture location	Femur (*n* = 25)	Tibia (*n* = 29)
Proximal, *n* (%)	15 (60.0)	3 (10.3)
Shaft, *n* (%)	5 (20.0)	10 (34.5)
Distal, *n* (%)	2 (8.0)	2 (6.9)
Hip dislocation, *n* (%)	3 (12.0)	
Ankle/pilon, *n* (%)		14 (48.3)

The SILENT test revealed an overall sensitivity of 75.0% (95%CI 64 to 84%) and an overall specificity of 99% (95%CI 97 to 100%) for pelvic/acetabular fractures and injuries of the lower extremity. The sensitivity was higher for detection of femur fractures (83%, 95%CI 61 to 95%) and for tibia fractures (93%, 95%CI 77 to 99%). The specificity is 99% independent of the region of interest (Table 3).

**Table 3:** Epidemiologic results of SILENT (point estimate and 95% confidence interval)

	SILENT overall	SILENT femur	SILENT tibia
Sensitivity	0.75 (0.64, 0.84)	0.83 (0.61, 0.95)	0.93 (0.77, 0.99)
Specificity	0.99 (0.97, 1.00)	0.99 (0.97, 1.00)	0.99 (0.98, 1.00)
Positive predictive value	0.94 (0.85, 0.98)	0.83 (0.61, 0.95)	0.93 (0.77, 0.99)
Negative predictive value	0.94 (0.91, 0.96)	0.99 (0.97, 1.00)	0.99 (0.98, 1.00)
Positive likelihood ratio	59.81 (22.41, 159.63)	65.88 (24.44, 177.60)	147.57 (36.94, 589.55)
Negative likelihood ratio	0.25 (0.17, 0.37)	0.18 (0.07, 0.43)	0.07 (0.02, 0.26)

**Fig. 3:**
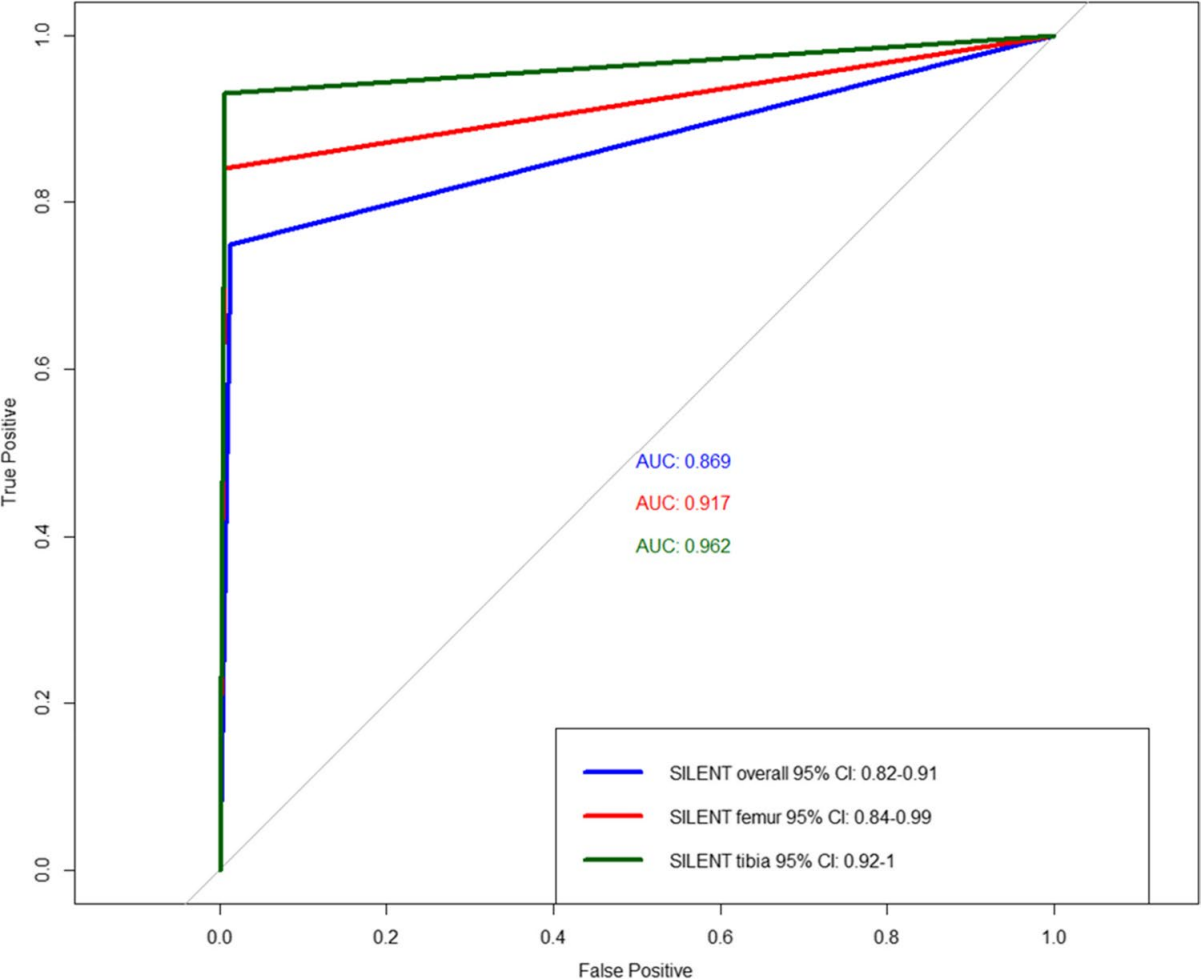
ROC and AUC analyses of the SILENT test with 95% confidence interval

In the intubated patient, the overall sensitivity of the SILENT test was 96% (95%CI 78 to 100%), and the specificity was 100% (Table 4). The prediction of an injury of the pelvis/acetabulum or the lower extremity revealed an AUC of 0.87 (95%CI 0.82 to 0.91). For femur fracture, the AUC was 0.91 (95%CI 0.84 to 0.99) and highest for tibia fractures (0.96, 95%CI 0.92 to 1.00) (Fig. 3).

**Table 4:** Epidemiologic results of SILENT in intubated patients (*n* = 34, GCS ≤ 8) (point estimate and 95% confidence interval)

	SILENT overall	SILENT femur	SILENT tibia
Sensitivity	0.96 (0.78, 1.00)	1.00 (0.66, 1.00)	0.86 (0.42, 1.00)
Specificity	1.00 (0.72, 1.00)	1.00 (0.72, 1.00)	1.00 (0.72, 1.00)
Positive predictive value	1.00 (0.85, 1.00)	1.00 (0.66, 1.00)	1.00 (0.54, 1.00)
Negative predictive value	0.92 (0.62, 1.00)	1.00 (0.72, 1.00)	0.92 (0.62, 1.00)
Positive likelihood ratio	NA	NA	NA
Negative likelihood ratio	NA	NA	NA

Twenty out of 336 negative SILENT tests were false negative (false negative rate 5.9%). Most patients had pelvic or acetabulum fractures (*n* = 13, 65%) or proximal femur fractures (*n* = 4, 20%). The remaining three patients had fractures of the medial malleolus *n* = 2, and avulsion fracture of the patella *n* = 1. These injuries were detected during a tertiary survey. Otherwise, no further fractures of the lower extremity were documented in the final discharge paper.

## Discussion

A rapid assessment of relevant injuries is essential in the initial evaluation of major trauma patients. Some clinical examinations are validated and represent a solid component of international accepted guidelines for the treatment of the major trauma patient [1, 13]. There still is a lack of a standardized clinical assessment tool for the evaluation of relevant injuries of the lower extremities. The clinical validation of the SILENT test revealed the following main results:

- The SILENT test is a standardized clinical assessment tool with a specificity of 99%
- The sensitivity of the SILENT test is highest for tibia fractures (93%)
- A negative SILENT test rules out femur shaft or tibia shaft fractures, not however pelvic/acetabulum fractures or fractures of the proximal femur

The SILENT tests can be performed during the primary survey at the patient’s arrival in the trauma bay [1]. The inspection of the undressed leg increases awareness of obvious fractures, bleedings, or open fractures. If the patient is conscious, the command to raise the leg assesses the present of major injury (SILENT) and serves as a part of the primary survey of disability. The present data show that the SILENT test is able to detect fractures of the tibia shaft and the femur shaft. The false negative rate is increased in the proximal femur fractures and pelvic/acetabular fractures. These injuries, however, are detected during the classic adjunct of the ATLS® (Pelvic X-ray) [2, 14] and are also included in the routine trauma CT scan. Therefore, the SILENT test is not recommended to test for the detection of pelvic, acetabulum, or proximal femur fractures during the primary survey. A negative SILENT test, however, rules out major injuries of the femur or the tibia. The SILENT test must not replace the tertiary survey. Minor fractures might still be diagnosed during the tertiary survey. The presented data show higher sensitivity of the SILENT test in the unconscious patient when compared with the conscious. The conscious patient might suffer from painful contusions of the lower extremity that might lead to a false positive SILENT test. An issue that is not reported in the unconscious patient. In comparison to other clinical adjunct, the SILENT test provides comparable results. It was reported that the FAST of the abdomen has a sensitivity of 74% and a specificity of 96% for intra-abdominal injuries [15]. An extension of the FAST (e-FAST) includes the detection of the pneumothorax [2] with a sensitivity of 78.6% and a specificity of 98.4% [16]. In comparison, a regular x-ray of the chest showed a sensitivity of 39.8% with the specificity of 99.3% for the diagnosis of the pneumothorax [16]. Van Leet et al. reported a sensitivity of 45% and a specificity of 93% of the prehospital examination of the pelvic ring regarding fractures [17]. A comparable study investigated a similar research question and reported a sensitivity of 31.6% and specificity of 92.2% regarding pelvic ring stability [18].

One might argue that the SILENT test could cause secondary damage to bones, soft tissues, and/or blood vessels/nerves. As pain serves as an indicator for a positive SILENT test, the risk for additional injuries is minimized. In the unconscious patient, the elevation of the leg is performed very gently to reduce the risk of secondary damages. The conventional palpation and crepitus detection-based clinical examination can be equally traumatic especially in larger, muscular extremities.

For junior members of the team in particular, a standardized clinical method for fracture diagnosis could be helpful in a potentially stressful environment of a trauma bay. It is important to notice that the SILENT test in the primary cannot replace a comprehensive and systematic musculoskeletal examination in the secondary survey. However, radiological imaging should not be performed until primary stabilization of the patient has been achieved. Further, the secondary survey after the primary stabilization of the patient is obligatory [2].

### Limitations

We are aware of certain limitations. One limitation would be the presence of ipsilateral fractures of the tibia and femur. It could be argued that detection of the femur fracture is not possible, as the test should be stopped after a positive result at the tibia, in order to avoid further damage to the soft tissue. Therefore, in cases of suspected multiple ipsilateral injuries, a whole-body CT scan including the foot, ankle, and phalanges should be performed on a low-threshold level. The decision to expand a trauma scan should be made after obtaining all necessary information from the physical examination. In the present cohort, hip dislocation, knee injuries, and fractures of the foot are underrepresented and might be subject to a type 2 error. The positive SILENT test is followed by a radiological examination that might rule out ipsilateral fractures. Second, one might argue that not all patients received radiological rule-out tests for fractures. The radiation exposure would not justify the performance of radiological imaging of all lower extremities, so the tertiary survey results and discharge summary were utilized to rule out missed fractures in the trauma bay. The documentation did not distinguish between the etiologies of negative SILENT test (visual, active elevation patient, elevation at the heel by the examiner). A further refinement was therefore not feasible. The SILENT test was not evaluated for the detection of fractures of tarsus/metatarsus or phalanges and should not be performed to rule out these types of fractures.

## Conclusion

The SILENT test detects major fractures of the femur shaft and the tibia shaft and can serve as a valuable clinical assessment tool during the primary survey of major trauma patients. A negative SILENT test rules out major injuries of the lower limb that are not included in the routine radiographic evaluation of the major trauma management. A positive SILENT test, therefore, warrants further radiographic assessments. Further large-scale prospective studies are required to assess the interobserver reliability of the SILENT test.

### Supplementary Information

Below is the link to the electronic supplementary material.

Supplementary file1 (DOCX 13 KB)

## Data Availability

The collected data will be stored securely in our institute for 10 years. During this period, they are still available upon request. After 10 years, the data will be deleted; however, all the datasets analyzed or generated during this study will be available from corresponding author upon reasonable request.
