# Supplementary material for: Clinical validation of the “Straight-Leg-Evaluation-Trauma-Test” (SILENT) as a rapid assessment tool for injuries of the lower extremity in trauma bay patients
Source: Eur J Trauma Emerg Surg. 2024 Jan 23;50(3):1119–25. doi: 10.1007/s00068-023-02437-z (PMC11249611; doi:10.1007/s00068-023-02437-z)
Supplement: Supplementary file 1 — Supplementary file1 (DOCX 13 KB) [file 68_2023_2437_MOESM1_ESM.docx]

SILENT-TEST

Li Bein: Re Bein:

Femur: Femur:

Knie-Instabilität: Knie-Instabilität:

Tibia: Tibia:

Example of a performed SILENT test documentation:

SILENT-TEST

Li Bein: Re Bein:

Femur: + Femur: -

Knie-Instabilität: - Knie-Instabilität: -

Tibia: - Tibia: -
